# A novel frame-shift mutation in *FRMD7* causes X-linked idiopathic congenital nystagmus in a Chinese family

**Published:** 2011-10-22

**Authors:** Wei Du, Juan Bu, Jiamei Dong, Yanlei Jia, Jing Li, Chen Liang, Shancheng Si, Lejin Wang

**Affiliations:** 1 Department of Ophthalmology, Peking University 3rd Hospital, Beijing, P.R. China; 2 Department of Cardiology, Party School of Central Committee of C.P.C., Beijing, P.R. China; 3 Department of Ophthalmology, The Municipal Hospital of ZaoZhuang, Shandong, P.R. China; 4 Department of Ophthalmology, Beijing Ji Shui Tan Hospital, Beijing, P.R. China

## Abstract

**Purpose:**

To screen mutations in the FERM domain-containing 7 (*FRMD7*) gene in a Chinese family with X-linked idiopathic congenital nystagmus (ICN).

**Methods:**

It has been reported that *FRMD7* mutations account for approximately 47% of X-linked nystagmus in Chinese patients. We collected 5 ml of blood samples from members of a family with X-linked ICN and 100 normal controls. Mutations in *FRMD7* were determined by sequencing PCR products.

**Results:**

We identified a previously unreported 4 bp deletion in *FRMD7* (c.1486–1489 del TTTT) in a Chinese family. The mutation co-segregated with the disease phenotype in patients and female carriers, while it was not detected in other relatives or in the 100 normal controls.

**Conclusions:**

Our results expand the spectrum of *FRMD7* mutations causing ICN, and further confirm the role of *FRMD7* in the pathogenesis of ICN. Direct sequencing of *FRMD7* could be used as a diagnostic testing of idiopathic congenital nystagmus.

## Introduction

Idiopathic congenital nystagmus (ICN) is primarily a disorder of neurologic control system that stabilizes the eyes defined as conjugated, spontaneous, and involuntary ocular oscillations. The symptoms appear at birth or during the first three months of life. Many of the patients show X-linked inheritance. Three disease loci of X-linked ICN have been mapped to chromosome Xq26.2, Xp11.4 and Xp22.3 [1-3]. In 2006, Tarpey et al. [1] reported a new member of the FERM family (4.1 protein, ezrin, radixin, moesin) that mapped to chromosome Xq26.2, FERM domain-containing 7 (*FRMD7*), which was associated with X-linked ICN. Mutations associated with ICN include missense mutations, null mutations, deletions, and frame-shift mutations [1,4-14]. Mutations in *FRMD7* are major causes of Chinese familial X-linked congenital nystagmus and account for approximately 47% of Chinese patients with the disorder [13].

*FRMD7* contains 12 exons and encodes a protein with 714 amino acids. In this study, we present a previously unreported mutation in the 12th exon of *FRMD7* in a Chinese family with ICN. We found a 4 bp deletion (c.1486–1489 del TTTT), resulting in a predicted truncation at amino acid residue 523. Our data expands on the spectrum of *FRMD7* mutations causing ICN, and further confirm the role of *FRMD7* in the pathogenesis of ICN.

## Methods

Clinical data and 5 ml of blood samples were collected from a Chinese Han family with ICN. The Institutional Review Board approved the project and investigators followed the principles of the Declaration of Helskinki. Informed consent was obtained from each person.

Human genomic DNA was isolated from blood lymphocytes according to standard protocol (Roche Diagnostics Corporation, Shanghai, China), using the DNA Isolation Kits for Mammalian Blood according to the manufacturer’s instructions (Roche Diagnostics Corporation, Indianapolis, IN). PCR-amplification of *FRMD7*’s 12 exons and exon-intron boundaries was performed using a standard 40 μl PCR buffer system with primers listed in Table 1. DNA sequence analysis was determined by BigDye™ terminator cycle sequencing with an ABI-3130 Genetic Analyzer (ABI Corporation, Carlsbad, CA).

**Table 1:** Primers used to amplify the exons of *FRMD7*.

**Exon**	**Forward primer**	**Reverse primer**	**Product length (bp)**
1	gctgagtttaagaaggctagagg	atttgctattgttgtcccttgag	563
2	aagggtaaatttgcagatgtagc	acaaagagggaggacaaaaactag	548
3	agggggcagattaaacgtag	gcagtgccagaaaatgagata	505
4	gaggggacggaagaggagagc	ggcataacccccaagtggatac	450
5	cccaaaaaggcatctgactg	aggccatgctgtttctctctatc	375
6,7	ccaaacacacacacccctatag	cctatttctgtccccatctatcc	851
8	Accccttcttgcttgcattc	ggcaaaagaaaagacacaccatc	440
9	ggagccaagtggaaaatcagaag	cccatcttcctccctcctagttag	480
10,11	gcgttctgagtagttgaggttgt	gccagttctctccagtctataagg	676
12,1	tctggaagtaggatggcattgag	tgattggctctgggacctttta	975
12,2	ccccaattagagcagaggaaagg	gccaacccatactgtcaccattc	962

## Results

**Figure 1:**
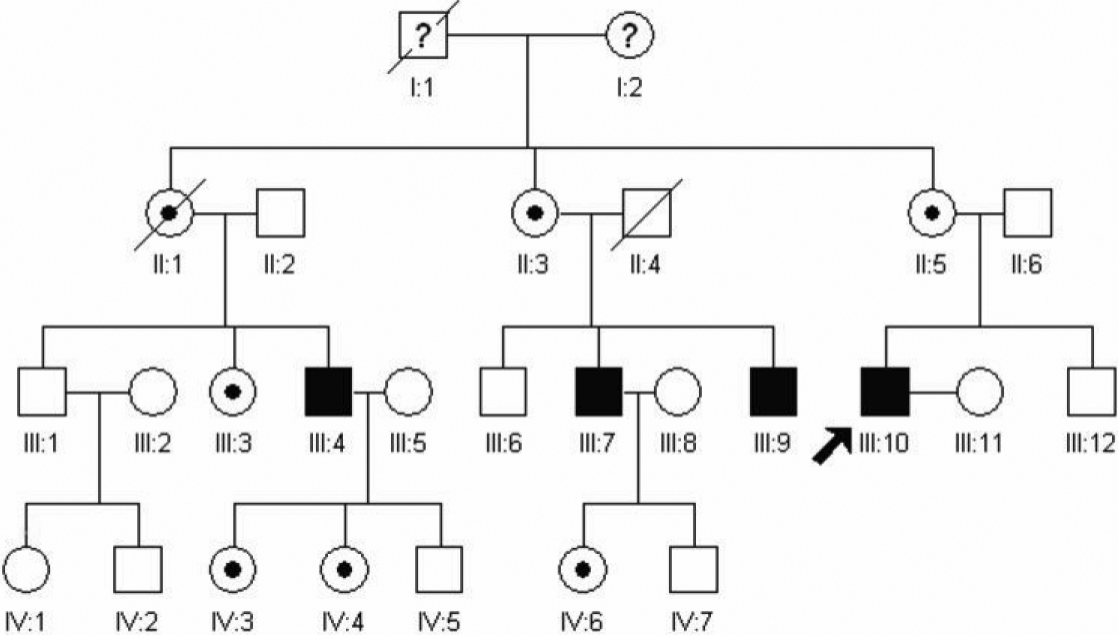
Pedigree of the Chinese family with ICN. The squares and circles represent males and females, respectively. The shaded symbols signify the affected individuals, the dotted circles represent female carriers, a diagonal line symbol indicates a deceased family member, and the arrow indicates the proband.

The family from Shandong Province, China, included 4 male patients and 5 female patients who were carriers (Figure 1). All patients in this family had various reduced visual acuity with a similar pattern of nystagmus.

**Figure 2:**
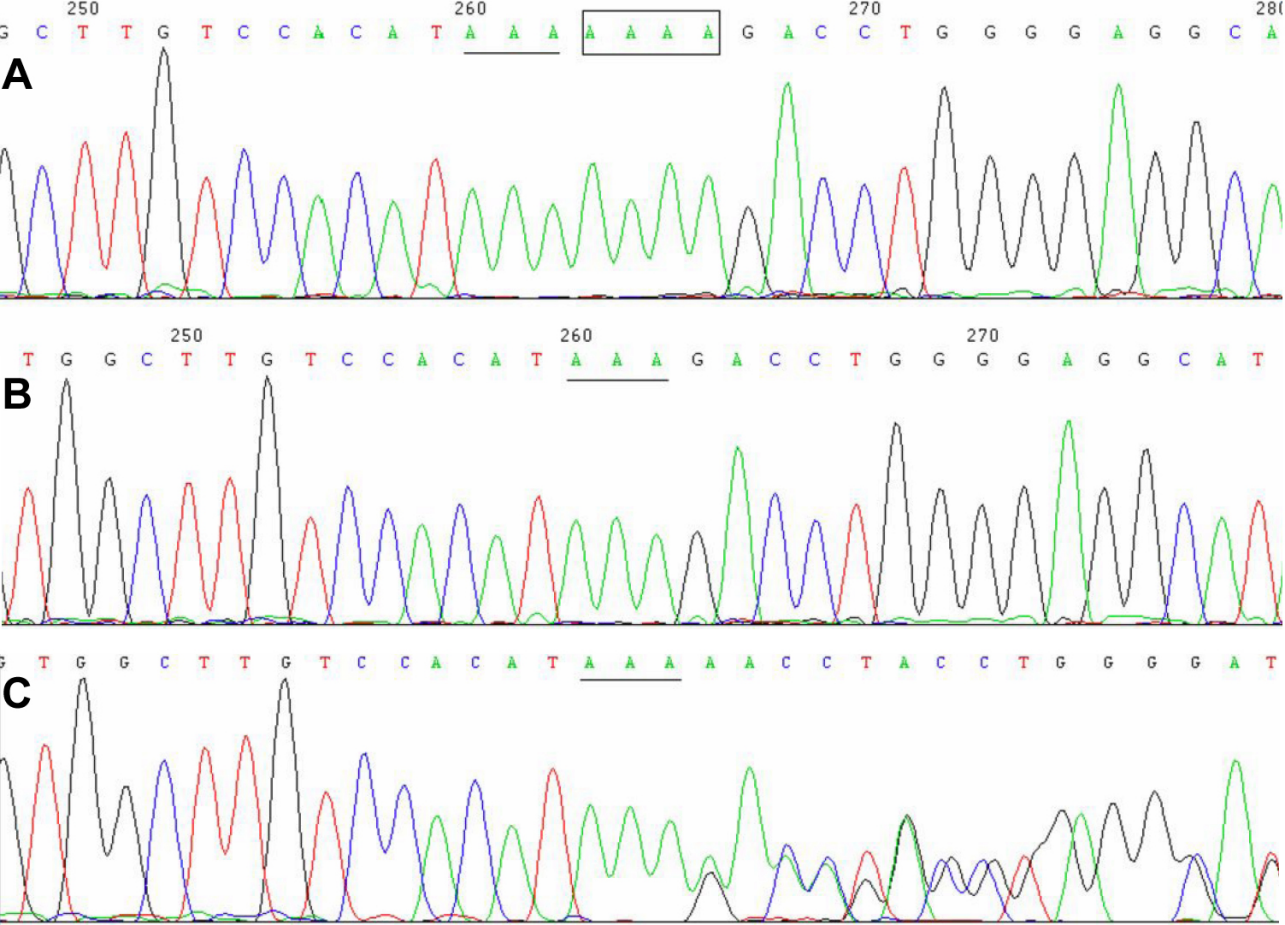
Sequencing chromatograms. **A**: Reverse sequencing chromatograms of a normal individual, **B**: an affected male, and **C**: a female carrier, showing a 4 bp deletion.

Sequencing of the 12 coding exons of *FRMD7* in one of the patients revealed a deletion in exon 12 (c.1486–1489delTTTT: Figure 2), which resulted in frame-shift at codon 497 and a putative stop codon 26 amino acids downstream in the translated protein (p.F497fs26X). The mutation was confirmed, and was further extended to other family members. The mutation co-segregated with the disease phenotype in male patients and heterozygous female individuals, while it was not found in other unaffected relatives or in the 100 normal controls.

## Discussion

FRMD7 shows a strong sequence homology with 2 other FERM family members, FERM, RhoGEF and pleckstrin domain protein 1 (FARP1) and FARP2, which are known to promote the dendritic growth of spinal motor neuron subtypes and modulate the length of neurite branching in developing cortical neurons, respectively [15-17]. Recently, Pu et al. [18] reported that expression of *FRMD7* in the fetal brain was mainly detected in the brainstem, which is associated with ocular motor control. Betts-Henderson et al. [19] found FRMD7 may play a role in multiple aspects of neuronal development.

**Figure 3:**
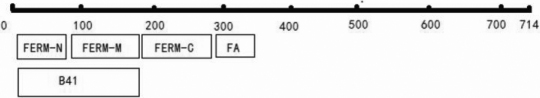
Graphic structure of FRMD7.

FRMD7 contains FERM-N, FERM-M, FERM-C, and FA structural domains in the NH_2_-terminus with conserved domains concentrated at the B41 and FERM-C domains (Figure 3). The B41 domain is located from residue 1 to 192, and the FERM-C domain is located from residue 186 to 279. Approximately 44 mutations causing congenital nystagmus have been reported in FRMD7, including 23 missense mutations, 8 splicing site mutations, 1 synonymous mutation, 4 nonsense mutations, 6 frame-shift mutations, and 2 deletions [1,4-14]. These mutations are mainly located in the FERM domain in FRMD7.

We are reporting the third frame-shift mutation in Chinese people. The presence of the mutation in all patients and carriers, and its absence in unaffected individuals and the 100 unrelated controls, support that the identified mutation causes the pathogenesis of ICN. The mutation identified in this study was found in the 12th exon, resulting in a truncated FRMD7 with FERM domain, while the COOH-terminus of FRMD7 protein was deleted. In the Pu et al. [18] study, a nonsense mutation type (COOH-terminally truncated protein) exhibited a different subcellular localization pattern from the wild type, which suggests that the COOH-terminus of FRMD7 may play a key role in the subcellular localization of FRMD7.

### 

#### Conclusion

Our results expand the spectrum of *FRMD7* mutations causing ICN and also confirm the role of *FRMD7* in the pathogenesis of ICN. Direct sequencing of *FRMD7* can be used as a method in gene diagnosis of idiopathic congenital nystagmus.
